# Allergic to Exercise: A Case of Exercise-Induced Urticaria and Angioedema

**DOI:** 10.7759/cureus.118496

**Published:** 2026-10-24

**Authors:** Puja Patel

**Affiliations:** 1 General Practice, National Health Service, London, GBR

**Keywords:** acute anaphylaxis, cholinergic urticaria, chronic inducible urticaria, exercise-induced anaphylaxis (eia), omalizumab

## Abstract

A 23-year-old female presented with urticaria, peri-orbital angioedema, wheeze and cough during intense exercise, with progression slowed by exercise cessation. Additional triggers included passive heating and strong emotion, while low-to-moderate intensity exercise did not provoke symptoms. Symptoms occurred independently of food intake. Past medical history included exercise-induced bronchoconstriction (EIB), eczema and seasonal allergies. Skin-prick and specific IgE testing for food allergens were negative, whilst dermatographia testing was positive. Specific IgE testing demonstrated sensitisation to house dust mite and animal dander as well as several moulds. Initial treatment with antihistamines, famotidine and montelukast provided partial symptom control, whilst later addition of omalizumab resulted in resolution of exercise-induced urticaria and angioedema.

This case illustrates the diagnostic overlap between exercise-induced anaphylaxis (EIA), a rare, life-threatening syndrome associated with exercise, and cholinergic urticaria, with a possible contribution from symptomatic dermographism and pre-existing symptoms of EIB. This case highlights that exercise-triggered urticarial syndromes may not fit neatly into a single diagnostic category, and that a thorough, trigger-focused history remains a valuable diagnostic tool.

## Introduction

Exercise-induced anaphylaxis (EIA) is a rare, life-threatening clinical syndrome that presents during or soon after exercise of different intensity [1]. Symptoms include urticaria, angioedema, flushing and gastrointestinal disturbance, with progression to airway compromise, hypotension and collapse [1,2]. Incidence is greater in young adults, affecting males and females equally, though it is more commonly seen in individuals with atopic conditions [1]. A nationwide survey of the United States population estimates that 1.5% of anaphylaxis cases can be attributed to exercise, based on the most recent reported episode [3]. Diagnosis of EIA is clinical, but definitive diagnosis can be challenging due to the overlap in presentation with other conditions triggered by heat or exercise, most notably cholinergic urticaria, a distinct form of chronic inducible urticaria (CIndU) [4,5]. Cholinergic urticaria is characterised by small urticarial wheals arising in response to a rise in core body temperature, whether from passive heating, strong emotion or exercise and can progress to anaphylaxis [4,5]. Management of EIA focuses on avoidance of activities which may provoke symptoms, supplemented by medication, although participation in exercise may be continued with adequate precautions [1]. These precautions include a gradual return to exercise, exercising with a partner and stopping exertion at the onset of symptoms [1,6]. Patients should be able to recognise the features of anaphylaxis, carry intramuscular epinephrine and be competent in its administration and contacting emergency services [1,6].

## Case presentation

A 23-year-old female presented with urticaria, peri-orbital angioedema, wheeze and cough with mucus production during intense cardiovascular exercise such as running. Symptoms began in 2018, typically starting with scalp pruritus followed by urticaria of the lower limbs (Figure 1) at sites of contact with clothing (buttocks, groin), spreading to areas uncovered by clothing including the face. If exercise continued this could result in peri-orbital pruritus and angioedema upon which exercise would be stopped. The time from the start of exercise to symptom onset was inconsistent, depending on exercise intensity rather than duration, and was not formally monitored by the patient. However, once scalp pruritus began, subsequent symptoms typically developed over five to 10 minutes. Cessation of exercise resulted in temporary worsening of symptoms for approximately 30 minutes, with eventual plateau alongside oral antihistamine use. Urticarial wheals were typically ≲5mm in size and resolved within 24 hours; however, angioedema could last 24-48 hours. Gastrointestinal symptoms, oropharyngeal swelling or syncope did not occur, and wheeze and cough were patient-reported, with no objective documentation of respiratory distress. Blood pressure was not self-recorded during episodes to establish whether hypotension was present, however there was no history of collapse. With prompt symptom recognition and cessation of exercise, symptoms did not progress to the point of requiring hospital admission.

**Figure 1:**
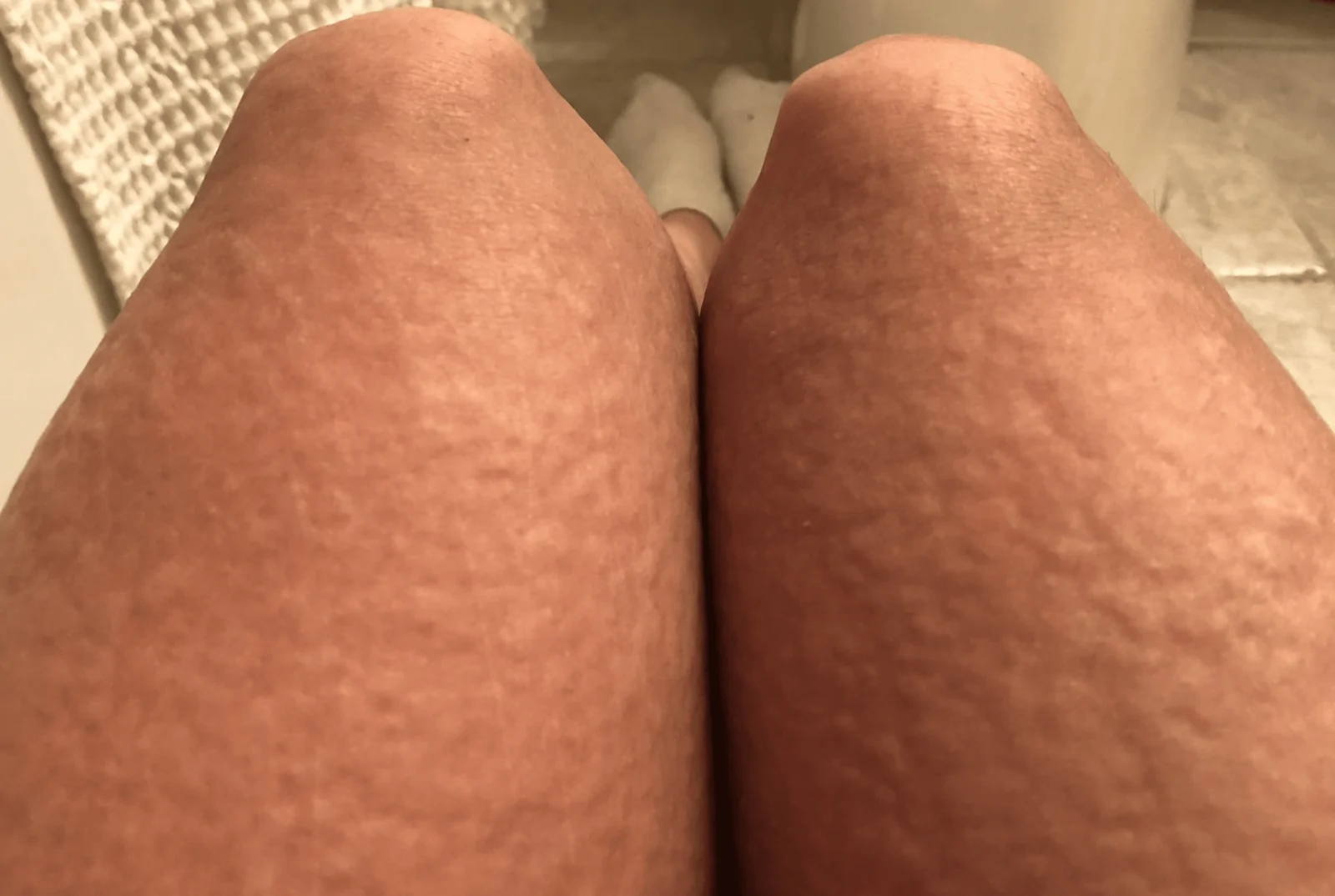
Excoriated urticaria affecting the legs following running.

In addition to exercise-dependent symptoms, urticaria could also be triggered by temperature changes such as prolonged hot showers, hot tub use, and emotional states such as embarrassment and anger. Notably symptoms did not occur with low-to-moderate intensity exercise such as walking or lifting weights. Symptoms occurred independently of food consumption prior to exercise and were unchanged when exercising in a fasted state (12 hours without food). She also reported rare episodes of urticaria confined to uncovered ankles in very cold weather (around 0˚C), without other symptoms. Symptoms occurred independently of seasonal changes and appeared unaffected by menstruation.

Past medical history was significant for exercise-induced bronchoconstriction (EIB), eczema, seasonal allergies and acne.

She was assessed by three different allergy and immunology specialist doctors, in 2018, 2020-2022 and 2023 onwards, the last of whom is her current treating physician. All patient encounters took place in outpatient clinics, with investigation results verified by the treating clinicians. Initial assessment in the year of onset (2018) included skin-prick allergy testing for food allergens. This was performed for apple, oat, wheat, potato, rice, spinach, sweet potato, peanut, chocolate, egg white and yolk, salmon, shrimp, catfish, lobster and cow’s milk, all of which were negative. Dermatographia testing in clinic was positive. Treatment at this time consisted of 5mg oral levocetirizine twice daily and famotidine 20mg taken 30 minutes prior to exercise. Inhaled albuterol (90-180 micrograms per dose) as needed was continued for this patient's pre-existing diagnosis of EIB. This regimen led to a reduction of exercise-induced symptoms, which could still occur with higher-intensity exercise and more prolonged heating. An epinephrine autoinjector was recommended alongside avoidance of high-intensity exercise and nonsteroidal anti-inflammatory drugs (NSAIDs), which were rarely used by the patient. Additional advice to exercise with a partner and stop exercise at the onset of any symptoms was given.

In 2020, two years into the course of her condition, famotidine 20mg was increased to 12-hourly on exercise days, with the first dose taken 30 minutes prior to exercise and levocetirizine was increased to 10mg every 12 hours due to refractory symptoms. All other medications remained unchanged. Due to patient-reported sedation with higher-dose famotidine and levocetirizine, only famotidine 20mg once daily and levocetirizine 5mg twice daily could be tolerated. Consequently, oral fexofenadine 180mg twice daily was trialled in place of levocetirizine.

On reassessment in 2022, a leukotriene receptor antagonist (LTRA), montelukast 10mg daily, was commenced due to persistent symptoms with exercise. Omalizumab was proposed by the treating clinician if the patient’s symptoms continued despite a trial of montelukast. Further tests at this time showed elevated total IgE (Table 1) and negative food-specific IgE levels (Table 2).

**Table 1:** Serum total IgE level result

Test	Result (kU/L)	Laboratory reference range
Serum total IgE	516	<214kU/L

**Table 2:** Food-specific IgE test results Class and interpretation of specific IgE levels (kU/L): Class 0 (<0.35) = Negative, Class 1 (0.35-0.70) = Low, Class 2 ( 0.71-3.50) Moderate, Class 3 (3.51-17.50) = High, Class 4 (17.51-50.00) = Very high, Class 5 (50.01-100.00) = Very high

Specific IgE	Serum IgE level (kU/L)	Classification of specific IgE (negative, low, moderate, high, very high)
Cow’s milk	0.25	Negative
Shrimp	0.11	Negative
Tuna	<0.10	Negative
Salmon	<0.10	Negative
Cod fish	<0.10	Negative
Scallop	<0.10	Negative
Wheat	<0.10	Negative
Soybean	<0.10	Negative
Cashew nut	<0.10	Negative
Peanut	<0.10	Negative
Hazelnut	<0.10	Negative
Walnut	<0.10	Negative
Almond	<0.10	Negative
Sesame	<0.10	Negative
Egg white	<0.10	Negative

During assessment in 2023, baseline serum tryptase was tested to assess the likelihood of mastocytosis, rather than excluding or confirming anaphylaxis. This was within normal limits (Table 3).

**Table 3:** Baseline serum tryptase result

Test	Result (µg/L)	Laboratory reference range (µg/L)
Serum tryptase	4.7	<10µg/L

Allergen-specific IgE testing was also performed; this showed sensitisation to several mould species and cat and dog dander (Table 4). The patient did experience sneezing, conjunctivitis and rhinorrhoea when exposed to dust, cats or dogs in keeping with allergic symptoms.

**Table 4:** Allergen-specific IgE results *olive, mulberry, oak, elm, alder, walnut, white ash, sycamore, cottonwood, birch, mountain juniper, acacia, box elder/maple, eucalyptus; † Bermuda, Timothy, Johnson; ‡ common ragweed, common pigweed, mugwort, Russian thistle, scale, lamb’s quarters, yellow dock, English plantain, cocklebur, firebush (*Kochia*) Class and interpretation of specific IgE levels (kUA/L): Class 0 (<0.10 kUA/L) = Negative; Class 0/1 (0.10–0.34) = Low; Class 1 (0.35–0.69) = Low; Class 2 (0.70–3.49) = Moderate; Class 3 (3.50–17.50) = High; Class 4 (17.60–50) = Very high; Class 5 (51–100) = Very high; Class 6 (>100) = Very high

Specific IgE	Serum IgE level (kUA/L)	Classification of specific IgE (low, moderate, high, very high)
Alternaria alternata	<10	Negative
Aspergillus fumigatus	0.16	Low
Aspergillus niger	0.19	Low
Penicillium chrysogenum	0.18	Low
Helminthosporium halodes	0.14	Low
*Dermatophagoides farinae* (American house dust mite)	19.10	Very high
*Dermatophagoides pteronyssinus* (European house dust mite)	9.87	High
German cockroach	0.22	Low
Cat dander	9.78	High
Dog dander	6.04	High
All trees tested (n=14)*	<0.10	Negative
All grasses tested (n=3)†	<0.10	Negative
All weeds tested (n=10) ‡	<0.10	Negative

Omalizumab is a monoclonal antibody that prevents binding of free IgE to high-affinity IgE receptors, often recommended where antihistamine and LTRA have inadequately controlled symptoms of chronic urticaria [5]. Omalizumab has been shown to reduce disease activity in antihistamine-refractory cholinergic urticaria [7], whilst an emerging body of case reports suggests it may also reduce exercise-induced symptoms in patients with EIA [2,8,9]. Monthly omalizumab 300mg injections were commenced in 2023, five years into the clinical course, owing to persistent symptoms despite montelukast and separate trials of levocetirizine and fexofenadine at fourfold standard dosing, which she also could not tolerate due to nasal and skin dryness.

This resulted in resolution of exercise-induced symptoms other than EIB; symptoms recurred when doses were missed. Current symptom control in 2026, eight years into the clinical course, has been achieved with ongoing omalizumab 300mg four-weekly, montelukast 10mg daily and fexofenadine 180mg taken one hour prior to exercise. High ambient temperatures are tolerated without symptoms, and whilst vigorous exercise is avoided, physical activity levels are maintained through low-to-moderate intensity exercise. With careful symptom monitoring, epinephrine has not been required to date.

## Discussion

EIA is a clinical diagnosis based on onset of symptoms during or within an hour of physical exertion. Specific criteria for anaphylaxis include acute onset of illness (minutes to hours) with skin or mucosal involvement (urticaria, angioedema, oropharyngeal swelling) with at least one of: respiratory compromise, hypotension or end-organ dysfunction (syncope, incontinence) or gastrointestinal disturbance (severe abdominal pain, vomiting) [10]. Alternatively, it is likely with any acute onset of hypotension, bronchoconstriction or laryngeal involvement following exposure to a known or likely allergen, regardless of skin involvement [10].

EIA may be food-dependent (FDEIA), with symptoms arising from exercise prior to or following ingestion of a specific food trigger, and notably, the same food does not provoke anaphylaxis when ingested in the absence of exercise or another cofactor [1]. FDEIA is considered to be related to IgE-mediated allergy, potentiated by increased intestinal permeability during exercise or other cofactors (alcohol, NSAIDs), resulting in increased food antigen absorption into the bloodstream [1]. This patient was suspected to have a potential diagnosis of EIA by the treating clinician in 2023, based on the onset of acute exercise-associated symptoms affecting the skin and respiratory system. FDEIA was considered less likely as symptoms occurred independently of prior food consumption, including after a 12-hour fast, and food-specific skin-prick and IgE testing were negative. With respect to EIA in this case, the presence of elevated serum tryptase or hypotension was not confirmed in line with features of anaphylaxis [10], and respiratory involvement (cough and wheeze) may have represented the patient’s pre-existing diagnosis of EIB rather than being a feature of EIA. Furthermore, the most common allergen with respect to FDEIA is wheat, specifically omega-5 gliadin, a component of gluten [11]. Measurement of omega-5-gliadin-specific IgE was not performed in this patient, but may have been valuable in identifying wheat-related FDEIA that may not be detected by skin-prick or standard IgE testing [6]. More extensive food allergen testing was not performed; consequently, FDEIA cannot be definitively excluded.

CIndU is characterised by the presence of wheals due to a physical trigger (dermographism, delayed-pressure, heat, cold, water, solar exposure or vibration) [5]. Cholinergic urticaria is a subset of CIndU triggered by a rise in core temperature, as seen during active heating (exercise), strong emotion or passive heating (hot showers or baths) [5,12]. This typically manifests with small (< 5mm) wheals within minutes of trigger exposure [5,6] and can progress to angioedema, bronchospasm and anaphylaxis [4,12]. In contrast, wheals in EIA are typically larger (10-15mm) [4]. Cholinergic urticaria represents a key differential diagnosis in this patient given symptoms triggered by strong emotion and passive heating in addition to exercise with small (typically ≲ 5mm) urticaria.

Exercise challenge testing with serum tryptase measurement following onset of anaphylaxis can support a diagnosis of EIA [1,4], with a peak tryptase typically occurring after one to two hours [10]. However, a negative result does not exclude it [1,4], and testing must be performed in a controlled setting given the potential for anaphylaxis [1]. As both EIA and cholinergic urticaria can be triggered by exercise, hot water immersion (passive heating) can be utilised to distinguish cholinergic urticaria [5]. No standard challenge testing was performed for this patient. Whilst both conditions carry a risk of progression towards anaphylaxis, requiring caution around exercise [12], challenge testing may hold additional value in determining the safety of returning to vigorous exercise.

Symptomatic dermographism is the most common CIndU subtype affecting 2%-5% of the population, which involves development of itchy urticaria on sites of friction [5]. Given the presence of a positive dermatographia test and initial distribution of urticaria during exercise at sites of friction (lower limbs, groin), this may have been implicated in the patient’s symptoms. Of note, many patients with CIndU exhibit more than one subtype concurrently [13], which may account for the development of symptoms at both friction and non-friction sites in this patient. The patient also reported rare, inconsistent episodes of urticaria affecting exposed ankles in cold weather. Given the infrequency, it is difficult to establish whether this reflected incidental chronic spontaneous urticaria or genuine cold urticaria [5] as a possible overlapping CIndU subtype; this was not formally tested for.

## Conclusions

This case highlights the diagnostic complexity of exercise-triggered urticarial syndromes, in which features from multiple conditions may overlap and coexist within a single patient. Distinguishing between these entities relies heavily on detailed clinical history, including the character, distribution and threshold of triggers rather than a single confirmatory investigation. In this patient, the dependence on high exercise intensity with progression to angioedema and respiratory compromise without a food-exercise relationship raises the possibility of EIA. Simultaneously, triggers of passive heat and emotional triggers support cholinergic urticaria as an important differential. The initial urticarial distribution at sites of friction, alongside a positive dermatographia test, further raises the possibility of a coexisting dermographism component. FDEIA was considered less likely by the treating clinician, though not fully excluded, on the basis of history and negative allergen-specific testing, highlighting the importance of this distinction in guiding safe exercise practices and avoiding unnecessary dietary restriction. The marked and sustained clinical response to omalizumab in this case reinforces the potential role of omalizumab as an effective treatment option for exercise-induced urticarial syndromes where there are potentially overlapping conditions and diagnostic uncertainty.
